# Mass-Suite: a novel open-source python package for high-resolution mass spectrometry data analysis

**DOI:** 10.1186/s13321-023-00741-9

**Published:** 2023-09-23

**Authors:** Ximin Hu, Derek Mar, Nozomi Suzuki, Bowei Zhang, Katherine T. Peter, David A. C. Beck, Edward P. Kolodziej

**Affiliations:** 1 Center for Urban Waters, University of Washington Tacoma, Tacoma, WA 98421 USA; 2 Department of Civil and Environmental Engineering, University of Washington, Seattle, WA 98195 USA; 3 Department of Material Science and Engineering, University of Washington, Seattle, WA 98195 USA; 4 Interdisciplinary Arts and Sciences, University of Washington Tacoma, Tacoma, WA 98421 USA; 5 Department of Chemical Engineering, University of Washington, Seattle, WA 98195 USA; 6 eScience Institute, University of Washington, Seattle, WA 98195 USA

**Keywords:** Non-targeted analysis, Mass spectrometry, Unsupervised machine learning, Source tracking, Source apportionment, Python

## Abstract

*Mass-Suite* (*MSS*) is a Python-based, open-source software package designed to analyze high-resolution mass spectrometry (HRMS)-based non-targeted analysis (NTA) data, particularly for water quality assessment and other environmental applications. *MSS* provides flexible, user-defined workflows for HRMS data processing and analysis, including both basic functions (e.g., feature extraction, data reduction, feature annotation, data visualization, and statistical analyses) and advanced exploratory data mining and predictive modeling capabilities that are not provided by currently available open-source software (e.g., unsupervised clustering analyses, a machine learning-based source tracking and apportionment tool). As a key advance, most core *MSS* functions are supported by machine learning algorithms (e.g., clustering algorithms and predictive modeling algorithms) to facilitate function accuracy and/or efficiency. *MSS* reliability was validated with mixed chemical standards of known composition, with 99.5% feature extraction accuracy and ~ 52% overlap of extracted features relative to other open-source software tools. Example user cases of laboratory data evaluation are provided to illustrate *MSS* functionalities and demonstrate reliability. *MSS* expands available HRMS data analysis workflows for water quality evaluation and environmental forensics, and is readily integrated with existing capabilities. As an open-source package, we anticipate further development of improved data analysis capabilities in collaboration with interested users.

**Graphical abstract:**

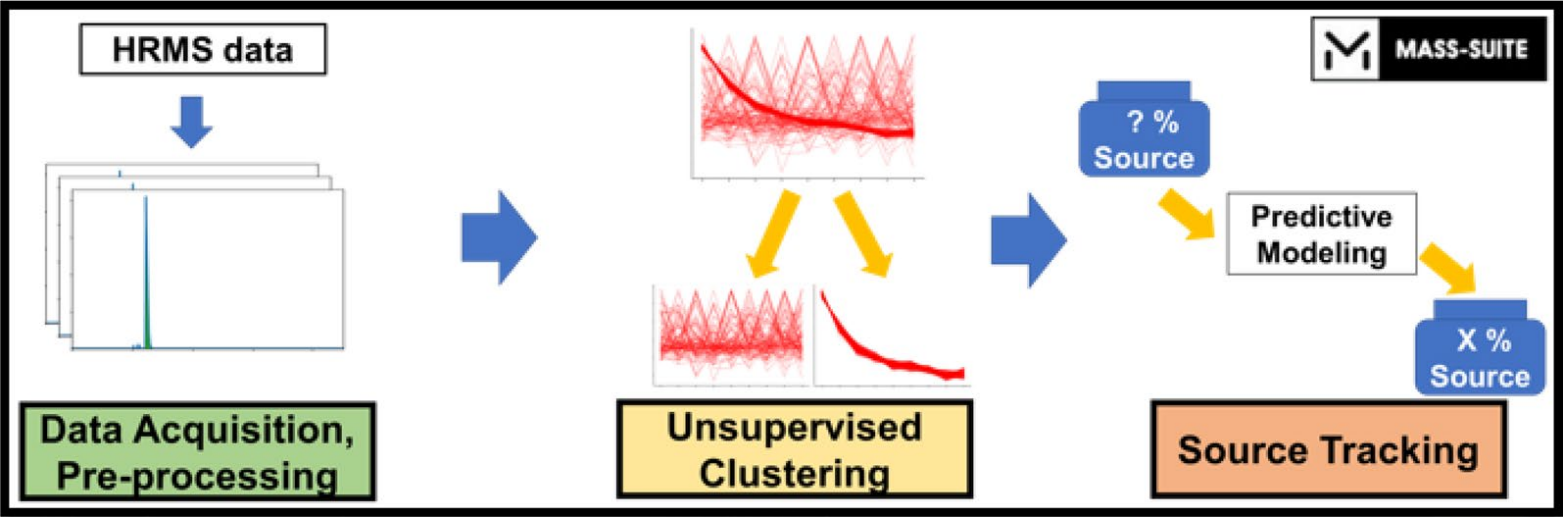

**Supplementary Information:**

The online version contains supplementary material available at 10.1186/s13321-023-00741-9.

## Introduction

High-resolution mass spectrometry (HRMS) analyses provide especially comprehensive and open-ended screening capabilities to characterize complex samples containing mixtures of many unknown or unanticipated compounds. With increasing recognition that humans produce and discharge many thousands of potential new “emerging contaminants” to the environment [1–3], these broad spectrum analytical methods are opening new frontiers in environmental chemistry, health, and engineering research. Specifically, non-targeted analysis (NTA) methods leverage the non-selective data collection capability of HRMS, with the resulting data supporting comprehensive characterization of chemical composition, identification of previously unknown contaminants, evaluation of compositional change across samples, and tracking of contaminant sources [4–9]. Such data uses are not unique to environmental analysis, with many applications relevant to multi-omics [10–12], toxicology, and drug screening studies [13–16].

Notably, pairing complex environmental samples with expansive HRMS data collection capacities results in generation of massive datasets; most such data remain under- or unused, in part due to limitations of existing data analysis workflows. HRMS data analysis workflows and software platforms incorporate data reduction, analysis, and interpretation elements, but significant opportunity remains for optimization and development of advanced data analysis capabilities, particularly for NTA data sets and for data interpretation endpoints beyond compound identification. Existing commercial software supports both basic data analysis (e.g., feature extraction, data alignment) and several advanced workflows (e.g., feature annotation, statistical analyses), but often are costly, limited to instrument-specific datafile formats, or provide outputs that struggle to interface with other platforms, databases, and tools. It is especially difficult for users to adapt existing workflows to integrate more complex approaches to feature prioritization, source tracking, or “-omics” analyses that require external functions or algorithms (e.g., machine learning, external database searching, or cloud computation). To address such needs, various open-source tools for handling HRMS data and implementing NTA workflows have been developed, including *MSDIAL* [17], *openMS* [18], *XCMS* [19], *MZmine* [20], *PatRoon* [21], and *enviMass* [22], among others. Those tools provide flexible workflows with designated functionalities within specific intended fields (e.g., proteomics or metabolomics), but often implement a limited range of data analysis capabilities (Table 1) or are not useful for some types of environmental data analysis (e.g., source apportionment).

**Table 1:** Overview of commonly used open-source software tools and their data analysis features for HRMS workflows

Features	Tools					
	*MSS*	TidyMS	MZmine2	XCMS^*^	MSDIAL	PatRoon
Language	Python	Python	Java	R	C#	R
Raw data preprocessing	√	√	√	√	√	√
QC-based batch correction	×	√	×	×	×	×
Quality reports	√	√	√	×	√	√
Normalization, imputation, scaling	√	√	√	×	√	√
Feature annotation	√	×	√	×	√	√
Isotope grouping	×	×	√	√	√	√
Interactive visualization plots	√	×	√	×	√	×
Clustering statistical analysis	√	×	×	×	×	×
Modeling tools	√	×	×	×	×	×

^*^XCMS is supported by various R packages and primarily acts as a starting point for subsequent analyses on other platforms

Raw HRMS data often consists of many thousands of features, requiring substantial computational resources and potentially driving inaccuracy in subsequent analyses if used directly. Therefore, feature filtering and prioritization are critical to effectively reduce the size of the dataset and facilitate downstream analyses [5, 6, 23, 24]. To avoid inefficient or impractical manual operations (e.g., to remove poorly integrated chromatogram peaks, to prioritize certain HRMS features) and facilitate data mining analysis, existing software platforms (e.g., Compound Discoverer, patRoon) commonly rely on descriptive statistics, data reduction, and data visualization (e.g., Principle Component Analysis (PCA), fold-change volcano plots) [21]. As a complementary automated approach, machine learning algorithms (e.g., supervised, unsupervised, or reinforcement learnings, etc.) can support more effective feature prioritization and predictive modeling workflows across several fields. For example, Nikolopoulou et al. developed a deep learning-based NTA workflow for environmental trend analysis to prioritize new emerging contraminants [25]. In metabolomics applications, machine learning algorithms can support clinical decisions, guide metabolic engineering, and facilitate biological studies [26–29]. However, existing workflows usually employ only one or a few algorithms concurrently, forcing users to jump back and forth between different platforms to achieve some analysis capabilities.

Currently, only a few software packages (e.g., *PatRoon*, *enviMass*) are specifically designed to address environmental NTA data analysis challenges. For example, identification and quantitative apportionment of complex chemical pollution sources remains a persistent challenge [4, 7]. Traditionally, contaminant source apportionment (i.e., estimating the presence and relative amount of a source in a mixed sample) has relied on the occurrence and quantification of a few pre-selected, targeted chemicals as unique source markers [30, 31]. However, source marker chemicals are not always known or unique to individual sources. HRMS datasets provide a unique opportunity to establish source “fingerprints” comprised of hundreds to thousands of both identified and unknown chemical features [4], which are more likely to be source-specific and to contain marker chemicals that persist through dilution and transformation processes. Conceptually, this approach enables complex mixture quantitation and represents an important, cutting-edge analytical capability [32–35]. However, few existing efforts have paired this concept with machine learning, indicating a clear opportunity for NTA workflows [7].

Finally, most open-source HRMS data tools were developed using R, C +  + , and Visual Basic programming languages, while relatively few software packages use Python [36–40]. As one of the most popular and accessible programming languages, Python especially benefits from community contributions, including the well-known statistical analysis packages SciPy [41] and scikit-learn [42]. Additionally, Python is an interpreted programming language that is relatively easy to read, learn, and write for non-programmer researchers, providing much flexibility and convenience for users to optimize and adapt existing tools to their needs [43].

Given these many data analysis needs and the limitations of existing software packages, we developed a Python package *Mass-Suite (MSS)* as an open-source data analysis toolbox with multiple HRMS data processing capabilities. The *MSS* package described here is compatible with exported data from other commercial or open-source tools and includes basic functions like feature extraction, prioritization, and data visualization. Driven by machine learning algorithms and capabilities that are not currently available within other NTA workflows or tools (Table 1), *MSS* also provides advanced data analysis (e.g., unsupervised clustering analysis, source tracking modeling), heuristic data exploration, data mining, and predictive modeling capabilities within a user-friendly, automated, and full-stack platform. We anticipate *MSS* will enable researchers, especially those with limited programming expertise, to more efficiently and reliably extract meaningful information from NTA datasets.

## Implementation

Development of *MSS* primarily depended on *Pandas* [44] and *scikit-learn* [42] packages for data processing and analysis, and *plotly* [45] and *matplotlib* [46] packages for data visualization. To demonstrate major *MSS* functionalities, this Implementation section describes a representative NTA workflow using *MSS* for data import, feature extraction and alignment, data reduction, advanced data mining (statistical analyses, feature clustering, and a novel source tracking function), data visualization, feature annotation, and reporting. In the Results and Discussion section, we describe workflow performance validation to assess peak picking accuracy and feature detection consistency relative to other open-source data processing platforms. Three example case studies are then provided to illustrate application of *MSS* to analyze existing experimental HRMS datasets. All related resources and an example workflow (in.ipynb format) are included in the demo file in the project GitHub repository: https://github.com/XiminHu/mass-suite. A README file accompanies *MSS* and most functions have individual documentation to ensure that *MSS* is readable and maintainable.

### Workflow development

**Fig. 1:**
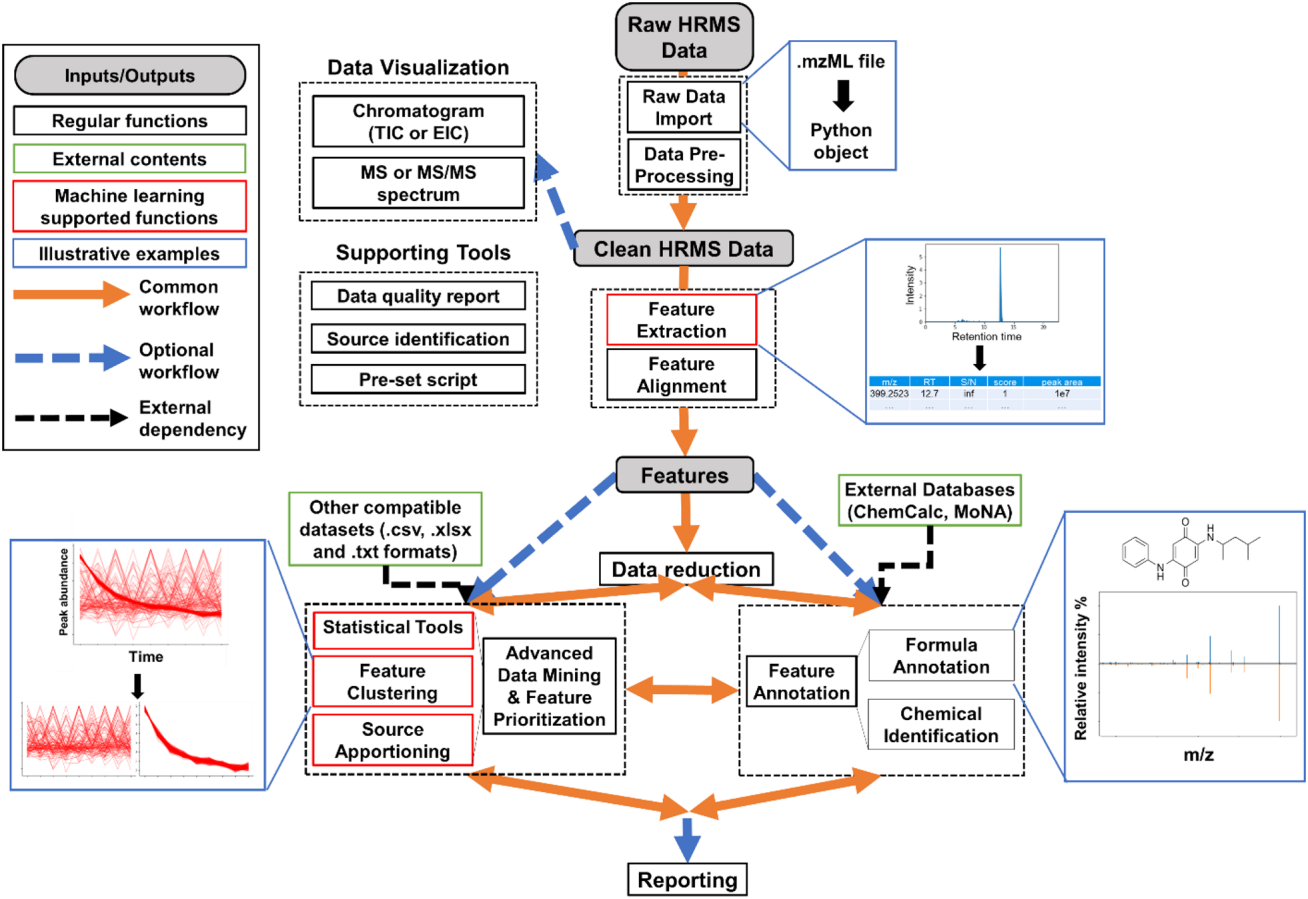
Overview of a typical *MSS* workflow for high-resolution mass spectrometry (HRMS) data analysis. The solid lines represent a typical workflow for typical HRMS non-targeted analysis (NTA) data processing; dashed lines represent additional optional workflows. All modules are optional

*MSS* uses a modularized layout to provide HRMS data analysis functions (Fig. 1) and all *MSS* modules can be loaded in full or separately as needed. Existing modules enable raw data import/pre-processing, feature extraction and alignment, data reduction, feature annotation, advanced data mining and feature prioritization, data visualization, and reporting. These functions and capabilities are summarized below and in Additional file 1: Table S1. All package modules are optional, customizable, and compatible with various external data formats (e.g.,.csv,.xlsx,.txt), enabling users to select and combine functions from different modules, external packages, and other platforms to create custom workflows.

For example, *MSS* capabilities can complement open-source software packages such as *OpenMS* [18], *XCMS* [19], *MetFrag* [47], *MSDIAL* [17], and *PatRoon* [21] by processing exported compatible output files with *MSS* functions. Users can also import.mzML files with the support of external data conversion tools (e.g., ProteoWizard [48], FragPipe [49]) to convert raw instrument-specific data formats (e.g.,.d,.raw). *MSS* is able to interface with external Python functions or packages, including several popular packages like *SciPy* [41] or advanced machine learning packages involving neural networks such as *TensorFlow* [50] and *PyTorch* [51]. New user-defined functions can easily be appended to existing modules to expand functionality and improve flexibility and data analysis capabilities. Statistical tools provided by *MSS* or *MSS*-interfaced external functions can process *MSS* data or external data in an Excel-compatible format.

### Data import, feature extraction, and feature alignment

In a typical *MSS* workflow, raw data (.mzML format) is first imported and parsed with *pymzml* [52] as Python-compatible metadata with the *mssmain.get_scans* function. Converted data are then available for optional baseline subtraction based on signal intensities prior to subsequent feature extraction (Additional file 1: Figure S1). HRMS feature extraction (i.e., “peak picking”), where a feature is a single presumptive detection of a chemical or its adducts/isotopologues and is represented as an exact mass (*m/z* value)—retention time (RT) pair, usually involves extensive parameter tuning and quality assessment of any extracted peaks. The *MSS* feature extraction function (*mssmain.peak_pick*) concatenates scans and finds peak indices (using the *PeakUtils* package [53]), then performs post-processing (e.g., peak width filter, replicated peak filter, regression-based peak boundary determination) to reduce poor quality features (e.g., peak splitting, insufficient scan numbers, high baseline noise). To further exclude noisy peaks, optimize parameters, and improve the feature extraction accuracy, *MSS* also calculates 15 descriptive parameters for the extracted peaks [54] to provide an optional peak assessment score based on a pre-trained random forest model. The complete feature extraction process for a single.mzML data file is described in Additional file 1: Text S1.

Because all parameters in this process are user-adjustable, the package provides options to trade off computational speed and feature extraction accuracy. For batch data processing of multiple.mzML files, the same workflow is performed on each datafile. After feature extraction, feature alignment across datafiles is performed based on Euclidean distance:

$${D}_{ij}= \sqrt{{{(mz}_{i}-{mz}_{j})}^{2}+{{(RT}_{i}-{RT}_{j})}^{2}}$$

where D_ij_ is the Euclidean distance between each feature observed in datafiles *i* and *j*, mz is the *m/*z ratio, and RT is retention time. Each feature (pair of *m/*z ratio and RT) that did not align with any existing detections would be used as a reference feature, and the aligned features’ *m/*z ratio and RT would be corrected to the same value as the reference features. Feature pairs with the lowest calculated distance across different datafiles are aligned. Aligned batch data can be exported as different user-defined formats (.csv,.tsv,.txt,.hdf, etc.) for subsequent analysis with other tools.

### Initial data reduction

Feature extraction and alignment usually yields datasets containing hundreds to thousands of HRMS features per sample. However, in NTA, more features do not necessarily indicate greater sample complexity and improved resolving power across samples, as internal (e.g., instrument/software artifacts) or external (e.g., background noise, impurities from sample processing) interferences may bias comparisons. Therefore, careful data reduction, supported by proper study design (e.g., experimental blanks, controls, replicates) to identify and exclude such interferences is important to ensure data quality and accuracy. Various customizable data reduction filters are available in *MSS* for HRMS feature lists. A representative data reduction process [21, 55] might include: (a) background feature subtraction based on a peak area fold-change criteria between experimental and blank samples; (b) replicate evaluation to remove features based on the calculated average and coefficient of variation for data from experimental or analytical replicates; and c) data trimming based on selected *m/z* or retention time ranges. These data reduction steps often effectively reduce feature numbers by up to tenfold, simplifying subsequent data analysis (*MSS* function example shown in Additional file 1: Figure S2). Although some “real” data is inevitably lost upon data reduction, stringent criteria for noise reduction and interfering detections typically improve the accuracy of downstream analyses, conserve calculation resources, and prioritize smaller data subsets for subsequent analysis [56].

### Advanced data mining

After initial workflow steps (e.g., feature extraction/alignment and data reduction), advanced analyses are often needed to extract meaningful information from NTA datasets [21]. When successful, these secondary data reduction processes also simplify the dataset and reduce the risk of incoherent classifications or predictions. Augmenting expected NTA workflow functionality, *MSS* provides basic statistical tools (e.g., hypothesis testing and trend comparison), as well as dimension reduction approaches (e.g., Principle Component Analysis (PCA) [57], t-distributed stochastic neighbor embedding (T-SNE) [58]) to reduce the “curse of dimensionality” [59] and provide simplified visualizations of complicated datasets. For example, PCA is easily performed using one-line commands in *MSS* (example provided in Additional file 1: Figure S3).

Beyond fundamental data mining tools, heuristic data exploration in *MSS* is supported by several machine learning-based approaches, including novel functionalities that are not offered by existing NTA workflows. These include: (a) clustering tools to aggregate features with similar behavior patterns (i.e., similar trends of normalized abundances) across samples based on unsupervised machine learning algorithms (such as density-based spatial clustering of applications with noise [DBSCAN] [60] and ordering points to identify the clustering structure [OPTICS] [61]); and b) a novel model-based source tracking tool. Detailed capabilities of these tools are described below.

### Feature clustering

In *MSS*, feature prioritization is performed by the unsupervised clustering algorithm DBSCAN by default, with OPTICS as an alternative algorithm. DBSCAN finds core features that possess high density, then expands clusters from these cores with cluster boundaries delineated by user-defined tolerances [62]. Compared to clustering algorithms commonly used in pattern recognition or temporal/spatial data grouping, such as KNN [63] and MeanShift [64], DBSCAN can discover clusters with arbitrary shapes, is robust towards outlier detections, and has been successfully utilized in various areas including biochemical studies and text processing [65, 66]. Using this approach, features with similar behavior patterns are automatically clustered with user-selected parameters, while outliers that diverge from recognized trends are excluded. Consequently, DBSCAN effectively prioritizes features that, for example, belong to a specific contamination source or are persistent or labile during a chemical reaction or treatment process. This can facilitate data processing and generate more accurate results, while avoiding the need for laborious manual data processing in conjunction with user-defined or custom workflows.

Clustering analysis is performed with the *MSS* function *dm.ms_cluster*, which uses Z-score data normalization prior to clustering by default to eliminate data skewness and kurtosis:

$$ z \, = \, \left( {x \, {-} \, \mu } \right) \, / \, \sigma $$

where z is z-score, x is feature peak area in the sample, μ is the average peak area of the feature across all samples, and σ is the standard deviation of the peak areas. Other normalization algorithms are available from user settings (e.g., 0–1 scale normalization, log transformation). Normalized datasets are then processed with the DBSCAN (or OPTICS) algorithm for feature clustering. Optional dimension reduction methods (PCA or T-SNE) are available in the function according to user needs. Two tunable parameters for the DBSCAN algorithm, *min_samples* and *eps*, are determined via feature numbers (*min_samples*) and knee plot (*eps*; *MSS* provides a function *eps_assess* for this process). Clustered results can be optionally visualized for output evaluation, cluster selection for modeling analysis (Additional file 1: Figure S4), and heuristic data exploration.

### Source tracking and apportionment

Leveraging the unsupervised clustering analysis in conjunction with predictive modeling approaches, *MSS* offers a novel source tracking functionality. In *MSS*, clustering functions described above are first used to isolate source fingerprints. Resulting fingerprint features are then aggregated to train and test a predictive source tracking model using user-selected algorithms (see Example III). A complete workflow for source apportionment prediction from a pre-processed dataset using the *MSS* function (*dm.feature_model*) includes:

1. Prioritization of source fingerprint features by clustering analysis: Clustering analysis is performed on the dataset to designate features that cluster with source-associated patterns (e.g., decreasing abundance with source dilution) as source fingerprint candidates. Proper experimental design and sample preparation methods (e.g., a dilution series of a pollutant source sample, samples differentially impacted by the same pollutant source) are required to identify and prioritize source-representative features.
2. Data treatment for model training: A subset of the pre-processed original data is selected based on the prioritized fingerprint candidates, converted into a function-compatible format (e.g., renaming, data transposition, etc.), and split into training and test sets.
3. Model training: Using pairs of detected abundance and known source concentration for feature(s) or feature cluster(s) of interest (e.g., single feature, grouped features from one or multiple clusters), the function trains the predictive model with user-selected algorithms. After training, the function optionally generates a performance report (e.g., coefficient of determination of the model [42]; visualized predicted vs. actual values) to support evaluation of the performance and importance of different feature clusters for accurate source apportionment.
4. Model validation and optimization: Trained models are validated using the testing data to assess model accuracy and avoid under- or overfitting. Based on the result of (3), users can tune model parameters or re-select feature cluster(s) to iteratively optimize results.
5. Source apportionment prediction: After model training and testing, users can deploy the model to evaluate source presence/concentration in unknown samples.

Currently, the *dm.feature_model* function incorporates several algorithms for multivariate regression, tree-based regression and support vector machine regression, providing flexibility for different datasets and user needs.

### Feature annotation

Feature annotation (e.g., assigning a specific chemical identity to a detected feature) in *MSS* primarily exploits external databases with web Application Programming Interfaces (APIs). *MSS* functions interface to the ChemCalc online calculation tool [67] for chemical formulae prediction and to the MassBank of North America database [68] for MS/MS fragment matching to facilitate identification (Additional file 1: Figure S5). For formula prediction in *MSS*, after candidate formulas are calculated from monoisotopic precursor mass by ChemCalc, prediction accuracy is evaluated with a dot-product based score via isotopic comparison between theoretical and observed spectra [17]. For compound identification, *MSS* supports individual or averaged spectra upload options and results retrieval, following MassBank database searching criteria and protocols. Processed data from *MSS* can be exported for further annotation using other platforms and databases, such as MetFrag [47], SIRIUS [69], GNPS [70], and NIST databases [71].

### Visualization, reporting, and user interface

Several visualization functionalities are available in *MSS* for HRMS data inspection (within the *visreader* module), including an overview *m/z* & RT scatter plot, total ion chromatogram (TIC), extracted ion chromatogram (EIC), and selected MS or MS/MS spectra. Raw HRMS data is inspected as the parsed list object (Additional file 1: Figure S6) or visualized using functions from the *visreader* module (Additional file 1: Figure S7). Output figures are available in static or interactive formats. Some visualization functions (EICs, MS and MS/MS spectra) provide optional online database search options and comparison with theoretical results (e.g., isotopologue pattern, MS/MS fragmentation) to help users understand and communicate HRMS data. Beyond designated visualization functions within the *visreader* module, data output visualization options are also integrated into most *MSS* functions, including those for advanced data mining (e.g., PCA, feature clustering analysis), for users to immediately evaluate package results.

*MSS* is designed to ensure easy interpretation and export of processed data. All processed data (as spreadsheets) can be saved with the *Pandas* function [44]. Visualization plots can be saved directly from the output window in user-defined formats (e.g.,.png,.jpg). Trained models for feature extraction and quantitative source apportionment can be serialized using the *pickle* package [72]. Recommended interfaces for *MSS* are through notebook-style integrated development environments either locally (e.g., jupyter notebook) or remotely (e.g., Google Colab), while feature extraction and data alignment functions can be executed as a command line script to allow running the software on a high performance computing cluster or the cloud.

### Software distribution and availability

MSS is distributed as a Python package with some external supporting packages developed with C +  + . The package currently supports *Microsoft Windows*, *Linux,* and *macOS* platforms. Documentation (https://github.com/XiminHu/mass-suite#readme) includes the latest patch notes, dependencies, tutorial examples, and example data for package testing. *MSS* was automatically tested during development with a continuous integration pipeline (GitHub Action). *MSS* distribution is generated with *dist* package and uploaded to PyPI server with *twine* package. Users can install the package via *pip install* command (https://pypi.org/project/mass-suite/), within Anaconda, or through the external command-line.

## Results and discussion

### Feature extraction reliability

**Fig. 2:**
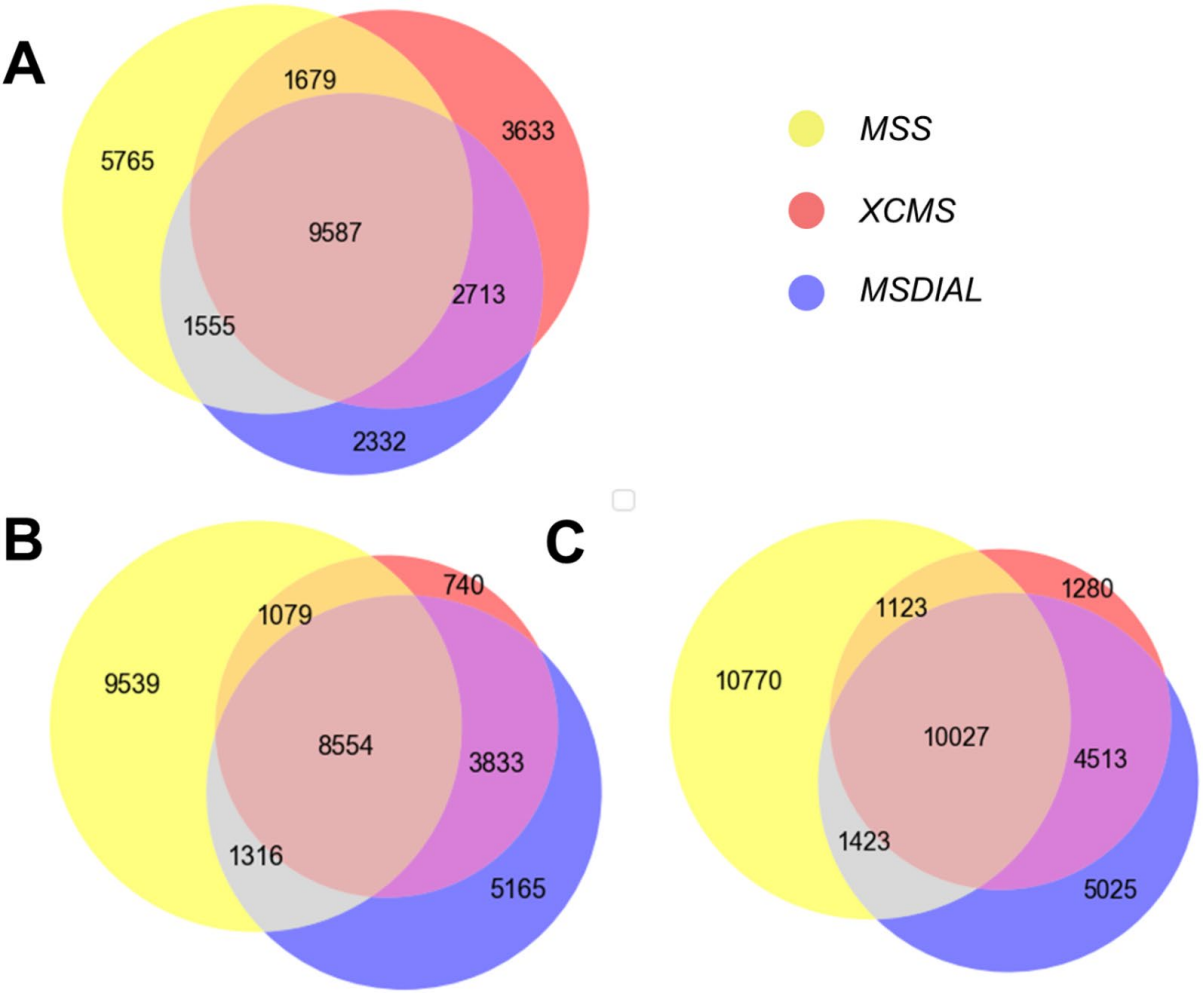
Comparisons of feature extraction outcomes for identical input samples. Samples numbered **A** #505, **B** #506 and **C** #508 from the ENTACT study [73] with *MSS*, *XCMS* and *MSDIAL* software processing. Venn diagrams report extracted features overlap between different platforms. The feature extractions were performed with parameters matched as closely as possible across the different platforms. Key parameters for peak extraction for different platforms are reported in Additional file 1: Table S2

In HRMS data analysis, manual inspection of all extracted chromatographic peaks is typically impractical, so feature extraction accuracy impacts the quality of subsequent analyses. To assess reliability of *MSS* feature extraction, archived samples (mixed chemical standards) from the EPA ENTACT study [73] (Additional file 1: Text S2; sample numbers #505, #506 and #508; 398 MS-amenable chemicals in total) were analyzed and processed through the *MSS* feature extraction workflow. The feature peak list was generated in *MSS* using default settings (Additional file 1: Text S3) and manually checked to validate correct extraction of chromatogram peaks for all chemical standards. *MSS* extracted 99.5% of peaks (2 peaks out of 398 didn’t match) known to be present in all three mixtures [73]. The extracted feature list from *MSS* for all archived ENTACT mixture samples (#505, #506 and #508) was then compared with two other open-source platforms (*MSDIAL* [17] and *XCMS* [19]) to evaluate feature extraction performance for total reported features (Additional file 1: Text S3). Most features extracted by *MSS* overlapped with those reported by other software (Fig. 2; on average 52 ± 5% and 52 ± 6%, for *MSDIAL* and *XCMS* respectively), validating *MSS* performance in comparison to other well-accepted feature extraction tools. RT & *m/z* differences between the overlapped features also suggested similar data processing outcomes across these three packages (Additional file 1: Figure S8).

### Multiprocessing benchmarks

To minimize computational runtime, multiprocessing is optionally available for the most calculation-intensive functions (*peak_pick* and *peak_list*) that handle single or batch-file peak extraction. Multiprocessing occupies all available cores for calculation by default but is user-customizable. The data files used for benchmarking were from the same samples (ENTACT #505, #506 and #508) as the feature extraction validation (Additional file 1: Text S3). Compared to single core processing, with all cores working, the processing time decreased from 201 ± 1.1 s to 58 ± 1.4 s (87 ± 3% of the theoretical maximum for 4 cores of computational power) for single file feature extraction and 897 ± 9.1 s to 350 ± 37 s (65 ± 7% of theoretical maximum) for multiple file (batch) feature extraction. Thus, parallel processing scripts did provide optional high-efficiency processing allowing for some optimization of computational resources.

### Demonstration of MSS applications

The sections above introduced *MSS* functionalities and described validation of the software package reliability. Here, three applications of *MSS* to analyze lab-generated datasets are provided, focusing on: I-II) automated feature prioritization and III) source tracking analysis. We note that *MSS* was not solely used for all HRMS data processing in examples I (feature extraction/alignment) and II (feature extraction/alignment, blank subtraction) to maintain consistency with other analyses and studies. All the detailed data processing processes were documented in the demo notebook in https://github.com/XiminHu/mass-suite/tree/master/DEMO (parameter settings would typically be different for each example, which are described in the following sections).

#### Example I: Clustering analysis to prioritize 6PPD transformation products

This example demonstrated use of the *MSS* feature prioritization workflow to facilitate non-target screening of transformation products from a reaction process. An early (pre-release) version of *MSS* was used to aid prioritization (by clustering analysis) and identification (by formula annotation) of potential transformation products of 6PPD (a tire rubber antioxidant) during laboratory ozonation studies, fully described in Hu et al. [74]. Initial feature extraction and data alignment in Hu et al. [74] was accomplished by *MSDIAL* (version 3.46) [17], with all subsequent data cleaning, formula annotation, and statistical analysis performed in *MSS* (pre-release version). The detailed data treatment method and parameter settings are found in Hu et al. [74]. Key outcomes were that the *MSS* data pre-processing workflow effectively reduced the total feature count across 61 unique samples (excluding blanks) from 41,808 to 936 by blank subtraction, replicate filtering, and intensity filters within desired *m/z* and RT ranges (*m/z* 100–900; RT 3–18 min). Clustering analysis in *MSS* with the DBSCAN algorithm (DBSCAN parameters: *eps* = *0.4, min_samples* = *3*) prioritized 297 features with trends of increasing peak area abundance during ozone exposure based on chemical clusters (processing time < 30 min). Ninety-eight features were retained after filtering based on detected abundance and predicted chemical formula; 9 features were eventually prioritized as potential 6PPD-derived and environmentally relevant TPs. Critically, the unique workflow provided by *MSS* allows users to discover clustered behavior patterns of HRMS features, select features with relevant patterns (e.g., increasing over time, as expected for stable transformation products), and reduce analysis time (compared to manual operation, typically ~ 15–20 h for a dataset of this size), thus facilitating feature prioritization.

#### Example II: Clustering analysis for biotransformation product discovery

To further demonstrate and validate *MSS* workflow capabilities for accurately prioritizing features of interest by clustering analysis, archived HRMS data obtained from a previous biotransformation study [75] was re-analyzed with *MSS* (version 1.1.2). Briefly, the synthetic progestins dienogest and drospirenone were incubated in batch reactors, with samples collected over time (0, 4, 10, and 29 h) to measure biotransformation kinetics and identify transformation products [75]. Initial feature extraction, data alignment, and blank subtraction used Agilent software (MassHunter Profinder (B.08.00) and Mass Profiler Professional (B.13.00). Originally, features were manually prioritized as potential transformation products based on molecular formula and diagnostic MS/MS fragments (~ 30 h manual time). Here, as an illustrative case, the data exported from the Agilent software (.csv format) was processed in *MSS* using clustering analysis to prioritize potential transformation products. *MSS* efficiently clustered features with similar trends (Additional file 1: Figure S4; DBSCAN parameters: *eps* = *0.3, min_samples* = *5*; total processing time < 30 min), with 18 features identified as potential transformation products from the input list (after pre-processing for blank and control subtraction) of 136 features. Among those, nine *MSS-*prioritized candidates matched products reported originally (dienogest: TP311, TP 309, TP327b; drospirenone: TP 384, TP 380, TP 370a, TP 370b, TP382c and TP 368), representing 82% of the 11 “major biotransformation products” reported in Zhao et al. [75]. The function was primarily tuned to prioritize potential TPs that were resistant to further reactions (i.e., monotonically increasing abundance). Thus, the manually-identified intermediate TPs (2 TPs, dienogest: TP 313; drospirenone: TP 364), which degraded after initial formation, were not reported in the *MSS* prioritization results. Note that changes to the parameter setting or search for clusters with smaller size would potentially allow the MSS algorithm to detect non-monotonically increasing feature clusters as well. While valuable, such efforts would require further optimization efforts to improve accuracy and exclude or reduce potential false positive detections. Overall, the *MSS* data reduction and clustering analysis workflow yielded accurate results and significantly reduced data processing time, with improved performance anticipated with further parameter optimization, additional feature information (e.g., MS/MS spectra), or additional data processing to reduce false positive and false negative results.

#### Example III: Source apportionment modeling

**Fig. 3:**
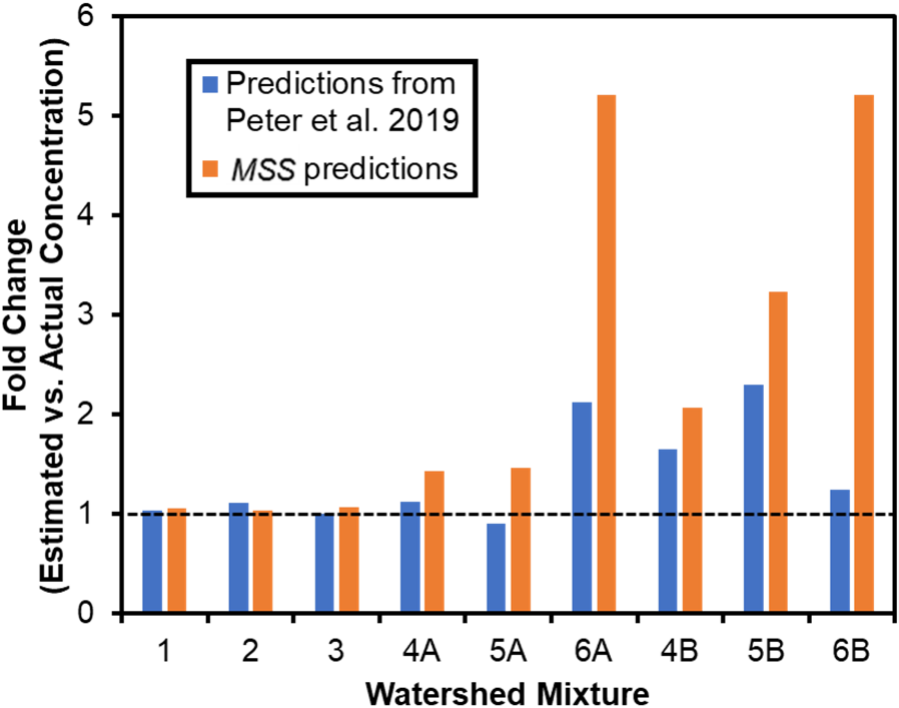
Estimates of fold change (estimated vs. actual concentration) of source (roadway runoff) concentration from a previous study [4] and *MSS* model predictions. *MSS* predictions were built from an ensemble random forest model that was trained with roadway runoff source sample dilution. One cluster of compounds (Cluster label = 0, N = 587) was prioritized from DBSCAN clustering analysis and used to derive estimates. The dashed line (fold change = 1) indicates predicted concentration equal to actual concentration

The source tracking approach within *MSS* builds on our previous laboratory study on this topic [4]; preliminary testing of *MSS* was conducted by re-analyzing archived sample data from that same study. Detailed sample composition and data acquisition methods are provided elsewhere [4]. Briefly, two complex roadway runoff samples were diluted and mixed with other water samples to mimic downstream mixing behaviors of multiple potential contaminant sources. In the original work, after HRMS analysis and data extraction using Agilent software (MassHunter Profinder (B.08.00) and Mass Profiler Professional (B.13.00)), fingerprint features were manually isolated and used to quantitatively apportion the amount of contaminant source in the mixed samples [4]. Using *MSS* (version 1.1.2; additional method details in Additional file 1: Text S4), we replicated this conceptual approach while incorporating machine learning approaches. HRMS source fingerprint features were isolated using a clustering analysis (DBSCAN parameters: *eps* = *0.6, min_samples* = *10*) of the diluted series of roadway runoff source samples. Subsequent model training, output summary, and source apportionment predictions are shown in Additional file 1: Figure S9. *MSS* predictions, using an ensemble random forest regression model (Additional file 1: Text S4), were compared with original prediction results (Fig. 3). Note that the *MSS* estimates were derived from an initial clustering analysis and model without further optimization, so accuracy could presumably be improved with iterative optimization. Challenges remain for improving predictions when a) limited chemical features are available at lower pollutant source concentrations (e.g., *MSS* prediction error ranged from 40 to 400% for Mixes 4A, 5A and 6A, which contained 4%, 1% and 0.6% pollutant source by volume, respectively, compared to Mixes 1–3 containing > 10% source); and b) co-occurring sources and/or the background matrix introduce features that overlap with source fingerprint features and bias predictions (e.g., *MSS* prediction errors were higher in mixes 4B, 5B, and 6B, which contain 10%, 2.5% and 0.4% by volume, respectively, of a second roadway runoff source). Nevertheless, prediction accuracy for mixtures with higher source concentrations (Mixes 1, 2 and 3; 30%, 18% and 10% pollutant source by volume, respectively) were similarly accurate (~ 5% differences in predicted source concentrations) as the original results, validating the utility of the source apportionment modeling function in *MSS*.

## Conclusions

We here communicated the structure and research capabilities of *MSS* as an open source and customizable HRMS data analysis software package developed with Python. *MSS* provides numerous default and user-defined modules (data import, feature extraction, data reduction, data visualization, feature annotation, and advanced data mining), that are accessible, flexible, and optimizable for custom study designs and data analysis scenarios, ensuring reproducible and accurate HRMS data analysis. Complementing traditional NTA data analysis approaches that focus on prioritization and identification of a small group of chemicals, core *MSS* functions provide a workflow for feature extraction, clustering analyses, and source tracking approaches that are supported by machine learning algorithms, allowing users to better leverage all relevant HRMS features for prioritization and modeling. These novel functions replace manual data reduction efforts and facilitate exploratory studies intended to utilize HRMS data as “big data”. While MSS provides functional documentation and examples for a quick and easy training guide for users with basic computational expertise, we do strongly encourage users to develop fundamental understanding about the algorithms, and their limitations and assumptions, that are used to generate results to avoid misinterpretation and misuse of the models. With respect to integrated software performance, the reliability tests and benchmarks also demonstrate the accuracy, efficiency, and power of *MSS* data analysis for various NTA and HRMS studies.

Because the *MSS* package is actively maintained and updated, to improve the coverage of different HRMS data processing need, e.g., feature grouping to merge the MS features (e.g., isotopes, adducts and in-source fragments) as individual chemicals. Additionally, several innovative functions and tools are in development for further NTA applications, including optimization of the chemical fingerprint-based source apportionment tool and a tool for matrix effect assessment and correction that leverages feature network analysis approaches. *MSS* is published on pypi.org, is fully open-source, and is available to anyone interested in using the default settings, adapting the code to their specific needs, or making contributions. Feedback and real-world case studies from interested users within the NTA community are especially welcome. We anticipate that the comprehensive, integrated functionalities provided by the *MSS* software package, together with its strengths of open availability, easy use, and external calculation resource compatibility will be especially useful to the HRMS and data science communities to assist with fully exploiting the rich datasets generated with HRMS instruments.

## Availability and requirements

Project name: Mass-Suite (*MSS*).

Project home page: https://github.com/XiminHu/mass-suite

Operating system(s): Platform independent (tested on Microsoft Windows and Linux).

Programming language(s): Python.

Other requirements: none.

License: MIT License.

Any restrictions to use by non-academics: none.

### Supplementary Information

**Additional file 1: **Additional experimental details, data processing methods, example code and output of the package.

**Additional file 2: **List of spiked chemicals for feature extraction validation samples.

## Data Availability

The datasets supporting the conclusions of this article are available in the mass-suite repository, https://github.com/XiminHu/mass-suite/tree/master/example_data.
