# Supplementary material for: Mass-Suite: a novel open-source python package for high-resolution mass spectrometry data analysis
Source: J Cheminform. 2023 Sep 23;15:87. doi: 10.1186/s13321-023-00741-9 (PMC10517472; doi:10.1186/s13321-023-00741-9)
Supplement: Supplementary file 1 — Additional file 1: Additional experimental details, data processing methods, example code and output of the package. [file 13321_2023_741_MOESM1_ESM.docx]

Additional file for

**Mass-Suite: A Novel Open-source Python Package for High-Resolution Mass Spectrometry Data Analysis**

Ximin Hu^1,2^, Derek Mar^3^, Nozomi Suzuki^3^, Bowei Zhang^3^, Katherine T. Peter^1,4^, David A. C. Beck^5,6*^, Edward P. Kolodziej^1,2,4*^

^1^Center for Urban Waters, Tacoma, WA, 98421, USA.

^2^Department of Civil and Environmental Engineering, University of Washington, Seattle, WA, 98195, USA.

^3^Department of Material Science and Engineering, University of Washington, Seattle, WA, 98195, USA.

^4^Interdisciplinary Arts and Sciences, University of Washington Tacoma, Tacoma WA, 98421, USA.

^5^Department of Chemical Engineering, University of Washington, Seattle, WA, 98195, USA.

^6^eScience institute, University of Washington, Seattle, WA, 98195, USA.

*Co-Corresponding authors

E-mail: dacb@uw.edu (D. A. C. Beck), koloj@uw.edu (E. P. Kolodziej)

**Additional file Table of Contents**

Text S1. *MSS* feature extraction process

Text S2. HRMS analysis for the ENTACT samples

Text S3. Data processing for the MSS function validation

Text S4. Data processing for example application I: source apportionment modeling

Figure S1. Example code and output for MSS data acquisition and parsing functions.

Figure S2. Example code and output for the data reduction.

Figure S3. Example code and output for the PCA statistical tool.

Figure S4. Example code and output for the DBSCAN clustering.

Figure S5. Example code and output for the feature annotation tool.

Figure S6. Example code and output for raw data inspection.

Figure S7. Example output for HRMS data visualization.

Figure S8. RT and m/z difference distribution of the aligned features between different software.

Figure S9. Example code and output for the modeling function.

Table S1. Major modules currently within *MSS* and related functions.

Table S2. Key parameters for peak extraction for *MSS, MSDIAL and XCMS*

Table S3. List of spiked chemicals for feature extraction validation samples (EPA ENTACT study) [1]

SI References

**Text S1**. *MSS* feature extraction process

The .mzML file would be parsed into python compatible object prior to the feature extraction process. An optional noise removal function is recommended to reduce the processing time and improve the feature extraction accuracy.

1. **Concatenate scans:** The closest neighbors (by *m/z*) of function-selected precursors (based on user defined mass ranges, error tolerances) from compiled scans are extracted with corresponding detection intensities. Extracted detections are aligned across all scans to compile a 1-D chromatogram intensity array for the proposed precursor mass. All precursor-associated 1-D arrays are subsequently merged as a chromatogram list.
2. **Find peak index:** Each precursor-associated 1-D chromatogram intensity array is processed individually. The peak indices within each array are located with the *PeakUtils* package, which finds the numerical index of the apex in the input data by taking the first-order difference of the intensity array [2].
3. **Determine peak boundaries:** After locating peak apex indices, peak boundaries are determined iteratively. The function traverses in two directions from the estimated peak apex while the boundary data points are iteratively checked that: a) for both left and right boundaries, signal intensity exceeds the user-defined relative baseline (defined as the ratio of boundary to apex intensities); and b) for the right boundary, every consecutive three points, if they didn’t meet the criteria in a), then a linear regression is performed with the three points and the next three consecutive points are checked only if calculated R^2^ value >0.6, otherwise, the third point is labeled a boundary point. Additionally, to exclude tails of prolonged peaks and reduce calculation time, peaks that surpass a user-defined maximum peak width are trimmed. After feature extraction, additional filters exclude duplicate detections (e.g., multiple noise peaks, overlapping detections). All numerical apex and peak boundary indices are then converted to scan time.
4. **Assess peak shape and remove noise:** Fifteen peak characterization parameters [3] are calculated during feature extraction. These parameters can be optionally screened by a pre-trained random-forest model for peak shape quality evaluation. The pre-trained model provided by *MSS* was trained with 5000 examples of manually labeled peak data and is available for re-training as needed. Peak shape was scored with values of 1-3 based on good, uncertain, and bad peak shapes, respectively, following published criteria [4].

To complete feature extraction, steps 2 to 4 are repeated for all possible precursors in the chromatogram list generated from raw HRMS data.

**Text S2**. HRMS analysis for the ENTACT samples

The instrument method was adapted from a previous study [5]. The methanolic samples (N=3; batch code assigned with the samples: #505, #506 and #508; 413 chemicals spiked in total) from ENTACT study [1] were provided by EPA (stored at -20 ℃). Details of the 398 MS-amenable chemicals are listed in **Table S3**. Constituents of all three samples were selected from ToxCast chemicals and verified to have >90% purity. #505 and #506 mixtures consist of chemicals that have no overlap in the monoisotopic mass to avoid erroneous identification. On the other hand, #508 mixture was developed with some isomeric and isobaric chemicals. All samples were analyzed using an Agilent 1290 Infinity ultrahigh performance liquid chromatograph (UHPLC) coupled to an Agilent 6530 quadrupole time-of-flight high-resolution mass spectrometer (QTOF-HRMS; Santa Clara, CA, USA). A reverse-phase C18 column (Agilent ZORBAX Eclipse Plus 2.1×100 mm, 1.8 µm) with a C18 guard column (2.1×5 mm, 1.8 µm) was used for the UHPLC separation at 45 ºC with 5 µL injection volume. LC separation used a gradient elution with mobile phases of 0.1% formic acid in each of deionized water (A) and methanol (B) as follows: 5% B at 0-1 min, 50% B at 4 min, 100% B at 17-20 min, 5% B at 20.1 min; stop time 22.5 min; post-time 2 min. The flow rate was 0.4 mL/min. Full scan data were acquired under 2 GHz Extended Dynamic Range mode at a range of 100-1700 m/z. Mass calibration was performed before the analysis.

**Text S3**. Data processing for the *MSS* function validation

After data collection, raw datasets were parsed and inspected for *m/z* and RT identifiers to confirm chemical presence. The datasets were also processed with *MSS* workflow with default settings (*noise_removal=2000, mz_error=10,* *peak_thres=0.001, peakutils_thres=0.1, min_d=1, peak_area_thres=1e5, min_scan=5, max_peak=5*; details in the function documentation). The retrieved peak list was compared with the manual confirmed *m/z* & RT list for the chemicals from the raw datasets. Among the 413 spiked chemicals, 398 were manually confirmed detection, while 25 chemicals excluded from analysis because of no detection. The alignment was performed between two lists for comparison, the closest neighbors from the two lists were screened with filters of *m/z* difference <0.015 and RT difference <0.5 minutes. Among the 398 detected chemicals, only 2 peaks were failed during the peak retrieval process. One chemical is m-Cumenyl methylcarbamate, due to multiple chromatogram peaks that potentially surpassed the maximum peak filter within the *peak_pick* function. Another chemical is Trinexapac-ethyl, which has a bad peak shape that likely contributed to the detection failure.

In addition to *MSS*, the MS datasets for samples #505, #506 and #508 were processed with MS-DIAL and XCMS [6, 7]. The data processing parameters were set as similar to those in *MSS* to the extent possible. For MS-DIAL: MS1 tolerance = 0.003 Da; Min Peak Height = 2000; Mass Slice Width = 0.01; Min Peak Width = 7 scan. For XCMS: MS1 tolerance = 10 ppm; Min Peak Width = 10 sec; Max Peak Width = 90 sec; S/N threshold = 10; MZdiff = 0.01; Prefilter Peaks = 1; Prefilter Intensity = 2000 (**Table S2**). All other default parameter values were retained. The feature peak lists generated with *MSS* were then compared to the peak lists from these two software platforms, using the same criteria for peak retrieval validation (*m/z* difference <0.015 and RT difference <0.5 minutes).

The multiprocessing benchmarks were performed on a personal laptop with pre-complied scrtips, which equipped with an 11th Gen Intel(R) Core (TM) i7-1165G7 @ 2.80GHz, 1.69 GHz processor (4 cores, 8 threads), 12 gigabyte RAM, SATA SSD storage, and Windows 10 Home operating system. The scripts were executed within PyCharm (version 2020.2.1) and the runtimes for peak extraction, with multiprocessing enabled and disabled using default parameters, were recorded in triplicate.

**Text S4**. Data processing for example application I: source apportionment modeling

The archived processed MS peak list in .csv format from a previous study [5] was used for the example application case. Initial data reduction of the imported data was done via *MSS*, to isolate features with maximum peak area > 100,000, *m/z* between 200-800 Da, retention time between 2-20 min, and present at peak area 5-fold greater than peak area in any of the solvent blanks or the ISTD controls. Features satisfying all these criteria were retained. After data reduction, DBSCAN clustering analysis was conducted to prioritize features as the fingerprint of the source (denoted “SR520”). All the peak areas were normalized as follows to eliminate data skewness and kurtosis:

z = (x – μ) / σ

where z is z-score, x is the peak area of the feature, μ is the average peak area of the feature across all the samples, and σ is the standard deviation of the peak areas. After normalization, the DBSCAN clustering was conducted on the SR520 dilution curve samples (*eps*=0.6, *min_samples*=10) to prioritize the features representative of the SR520 source. Three clusters were automatically populated (Cluster 0, 1 and 2; N=630 in total) and used for model training. The data were randomly split as training and testing datasets and an ensemble random forest model was trained and validated using the 630 features prioritized. After modeling, the model performance report was generated based on the coefficient of determination. Based on the model score, cluster 0 (score=0.993; N=587) was used to estimate the source concentration of the mixture samples (**Figure S9**).


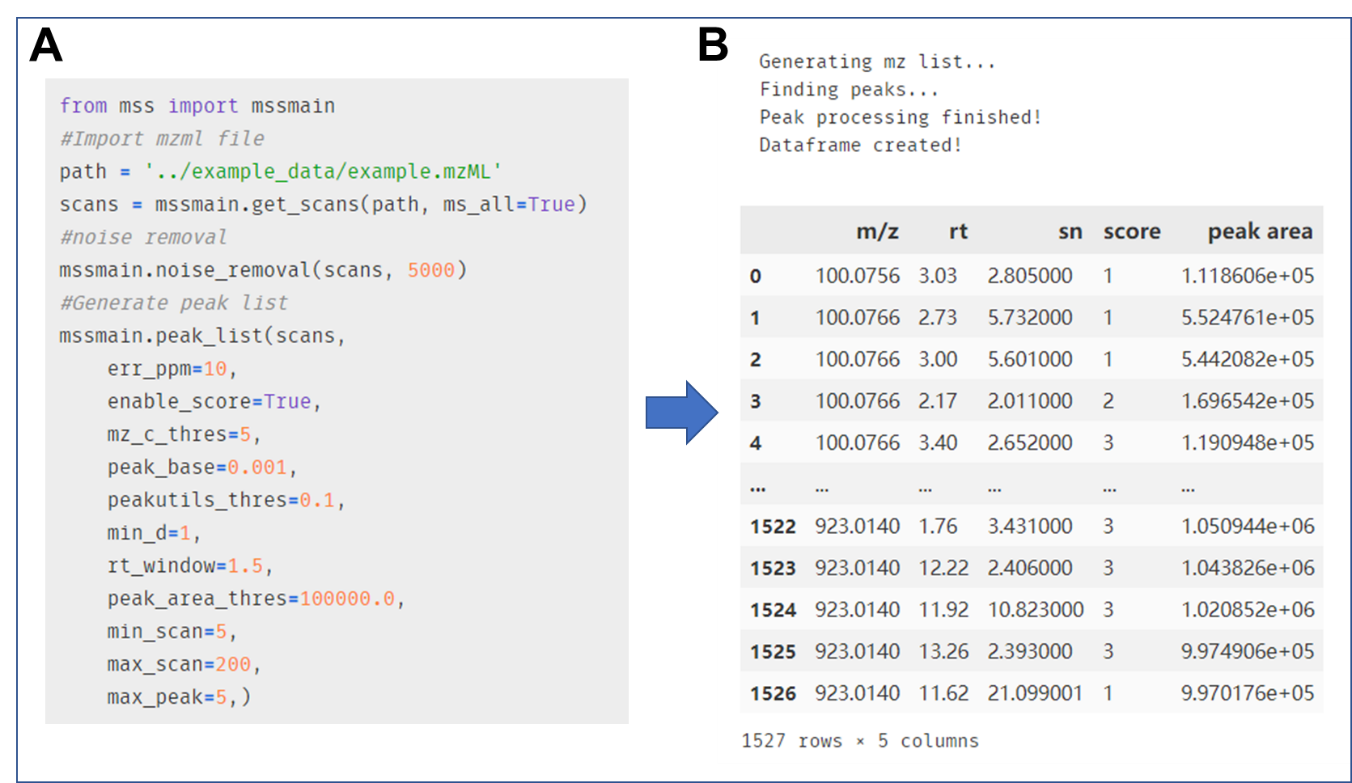


**Figure S1**. Example code and output for *MSS* data acquisition and parsing functions. (A) Example code for .mzML file reading, noise removal and peak list retrieval. All the parameters shown in the example code are default parameters. (B) Example output from the single file processing function *peak_list*. The score of the peak was calculated based on the built-in random forest model within *MSS*.


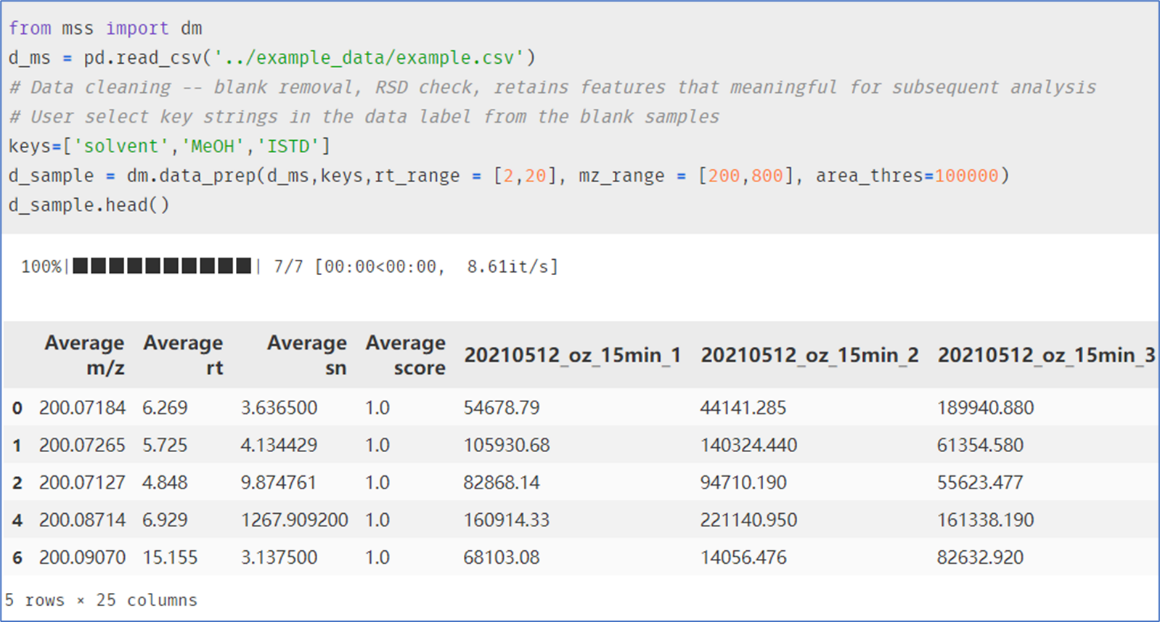


**Figure S2**. Example code and output for data reduction functions. The example data were obtained from a previous study [8]. For the data reduction, *MSS* relies on user-defined restrictions (*rt_range, mz_range* and *area_thres*) and performs blank subtracttion according to the *keys* statement. The *MSS* data reduction function also provides a consolidation option where replicates of the sample data will be automatically averaged and *MSS* will calculate the coefficient of variance for each averaged sample datapoint.


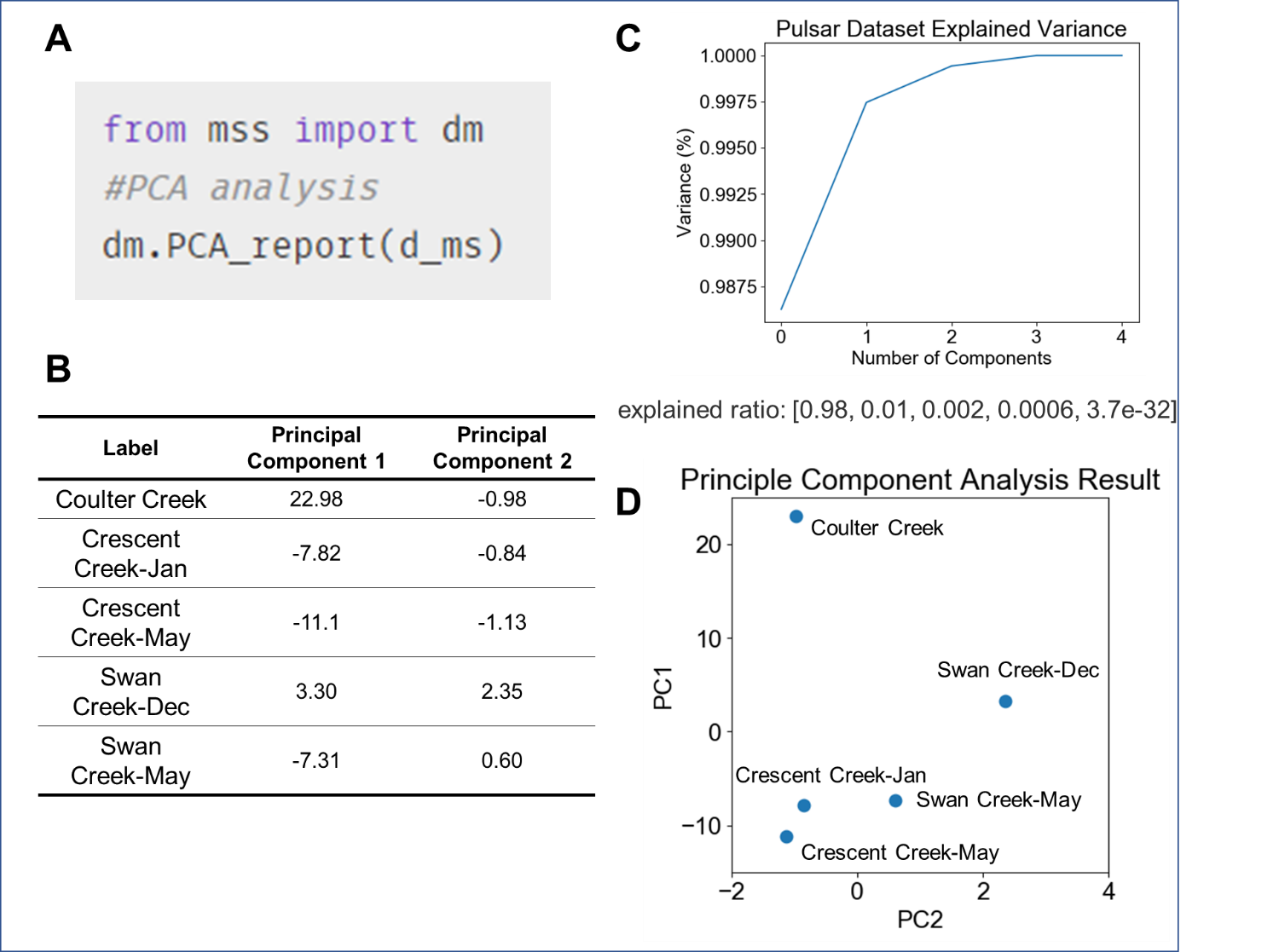


**Figure S3**. Example code and output for the PCA statistical tool, using archived experimental data from a previous study [5]. Each datapoint was calculated as the mean of triplicate experimental data; labels for each datapoint represent the sampling site and time (if the sites were sampled more than once). (A) Example one-line code for executing PCA analysis. The d_ms is a dataframe pre-populated within previous codes (**Figure S2**). (B) Summary of principal components for the averaged datapoints from samples with different ozonation reaction time. (C) Cumulative explained variance vs. number of components. (D) PCA figure for the 1^st^ and 2^nd^ principal components for visualization. The PCA result evaluated chemical composition similarity between different creeks or the same creek at different sampling dates.


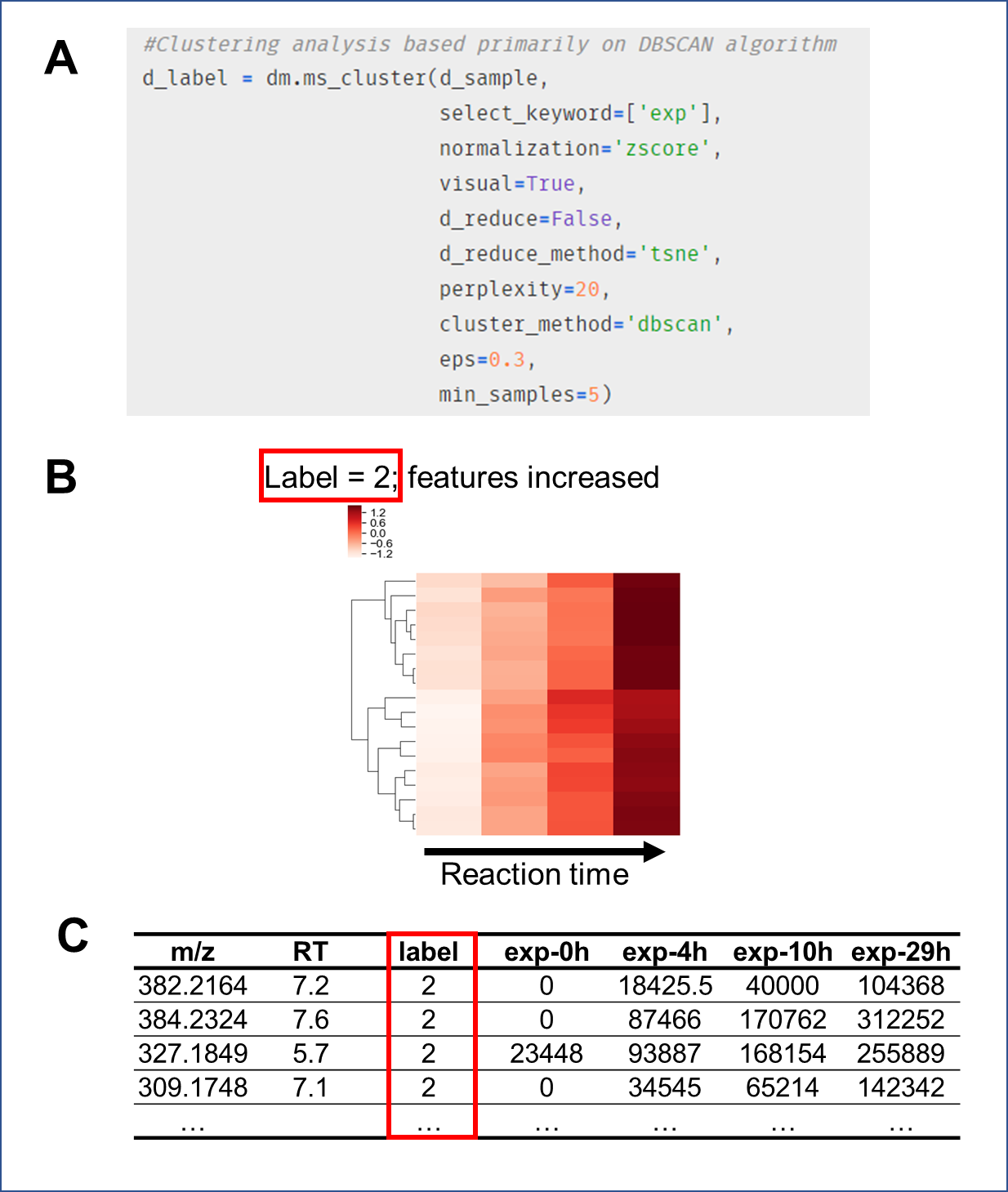


**Figure S4**. Example code and output for the DBSCAN clustering tool applied to the synthetic progestin biotransformation dataset [9]. The data in .csv format was processed with following settings using DBSCAN algorithm (*eps=0.3, min_samples=5*). (A) Example code for deploying DBSCAN. (B) Example heatmaps for the DBSCAN grouped clusters for data evaluation and inspection during the NTA process: the labels represent the group number of the cluster and the color represents feature detected abundance (peak area). The feature clusters generated from the function were manually selected to prioritize the clusters that potentially consist of transformation products. For example, the label 2 cluster contains features that increased during the process, suggesting potential reaction products. (C) Example processed dataset with the cluster labels column (highlighted with red boxes), this data subset could be prioritized based on the clustering label for further analysis (annotation, source tracking, etc.).


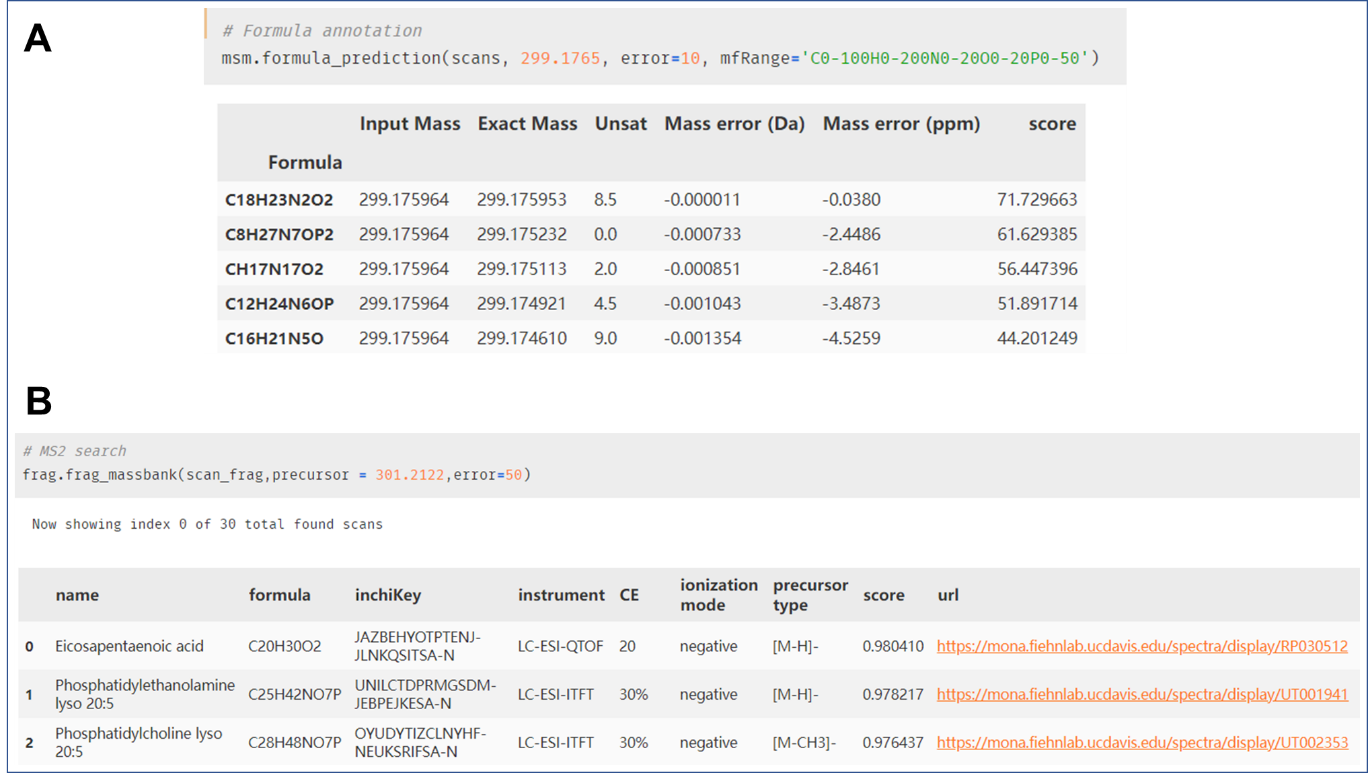


**Figure S5**. Example code and output for the feature annotation function. (A) Example code and output for the formula calculation function. The formula calculation is based on ChemCalc API [10]. (B) Example code and output for the MS2 online search annotation tool are based on the MassBank North America database [11].


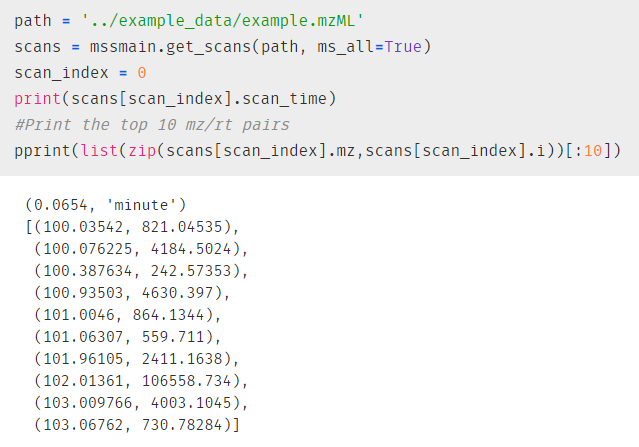


**Figure S6**. Example code and output for raw data inspection. After data was acquired and converted by *MSS*, the .mzML data could be interfaced directly as a python-list object.


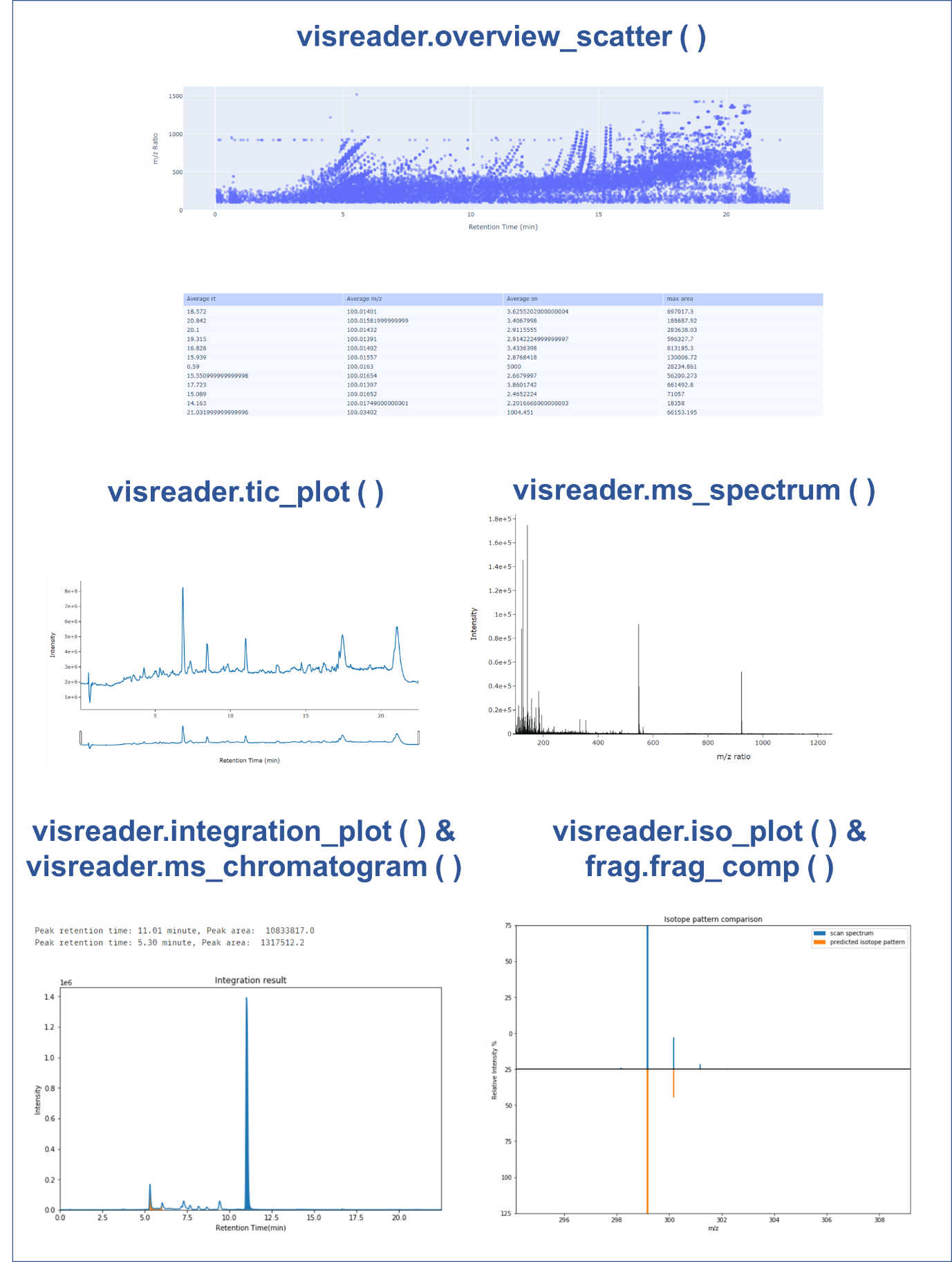


**Figure S7**. Example output for HRMS data visualization. The *overview_scatter* function is plotting all the extracted features from the *MSS* generated peak list and assists users in evaluating the feature extraction and sample data quality. The *tic_plot, ms_spectrum, integration_plot* and *ms_chromatogram* functions are used for raw data inspection, as assistance to existing software or workflow. The *iso_plot* and *frag_comp* functions are accompanied with feature annotation functions, users could visualize the MS spectrums for isotopic pattern comparison or MS/MS spectrums for chemical identification, using online or in-house databases.


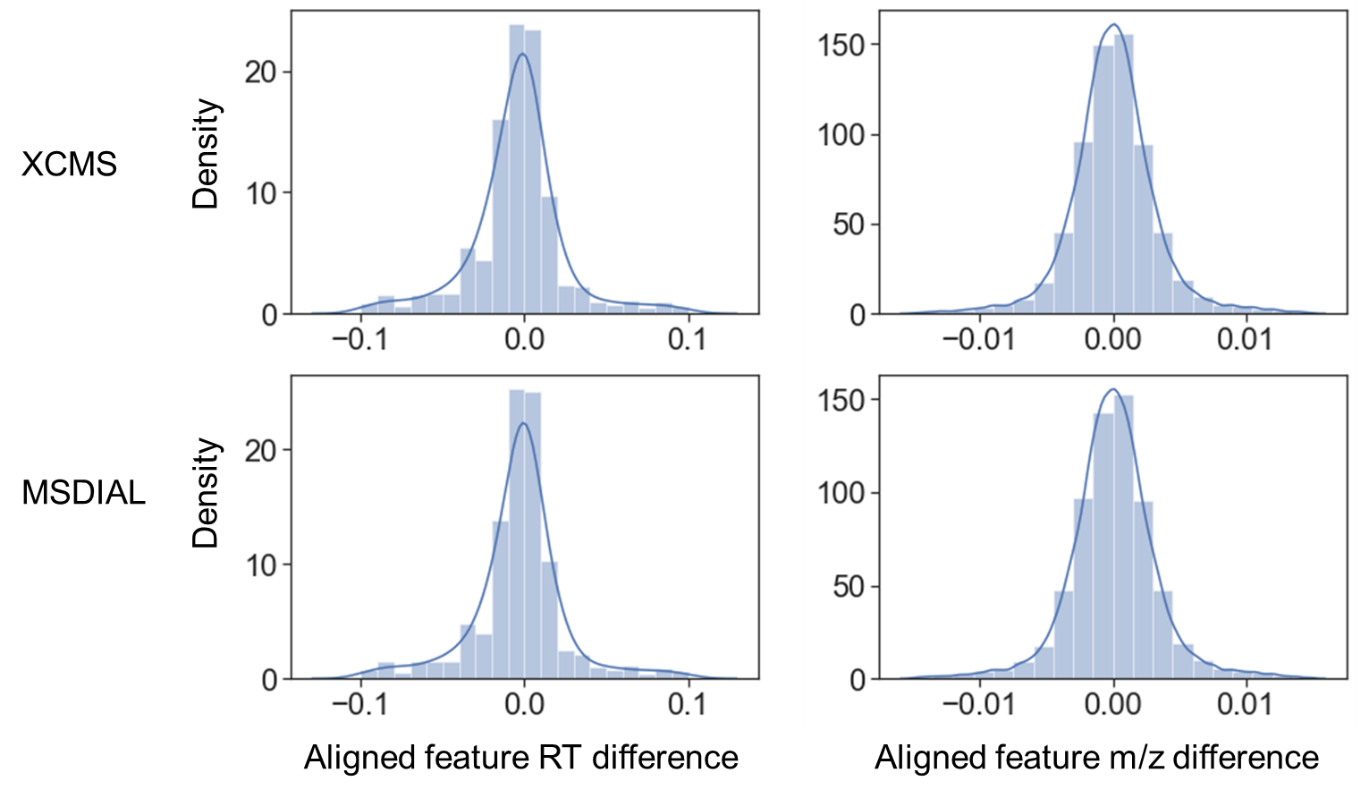


**Figure S8**. RT and *m/z* difference distribution of the aligned features between different software. The comparisons were separately conducted for *XCMS* or *MSDIAL*, each relative to *MSS*. All the RT and *m/z* values from MSS-extracted features were subtracted from the corresponding values from its closest neighbor (closest *m/z* and RT pair, determined by Euclidian distance) in *XCMS* or *MSDIAL* for comparison. The comparison results for RT and *m/z* differences from all 3 testing samples (ENTACT #505, #506 and #508) were integrated. The hist bins represent the original data, while the curves are kernel density estimation curves that represent probability densities calculated from the original data.


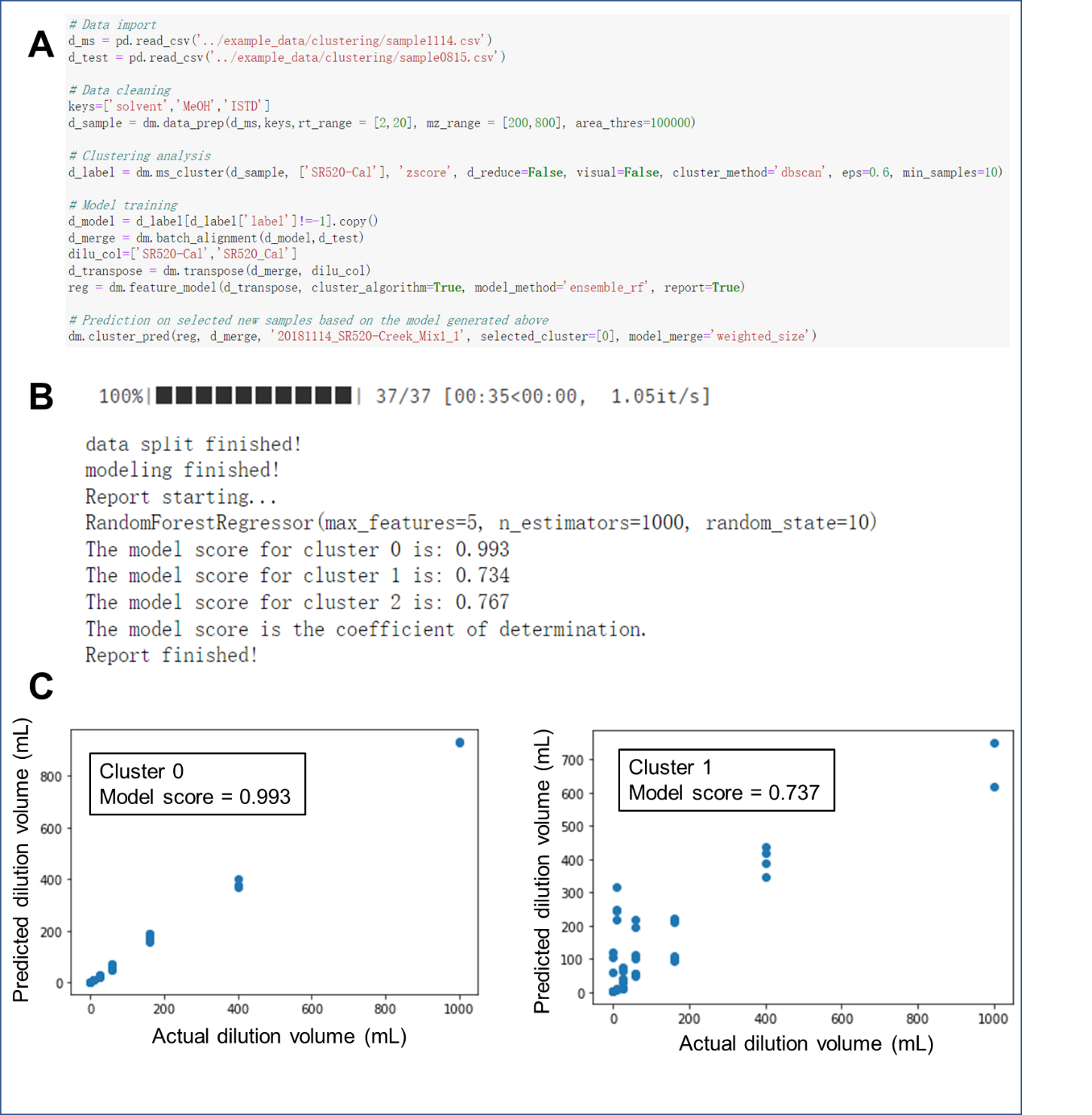


**Figure S9**. Illustrative example code and output for the modeling function. Archived sample data from a previous study were re-analyzed for method development and testing [5]. A) example code for the data reduction, feature clustering, model training, model evaluation and source concentration prediction. Data reduction was performed with following settings: *RT*=2-20 min, *m/z*=200-800, *Peak Area*>100000. DBSCAN algorithm (*eps*=0.6, *min_samples*=10) was used to cluster features for subsequent modeling functions. B) Example output for model performance evaluation and dilution volume prediction. The ensemble random forest model was trained with data subsets of selected features (#0). The model performance report is available for users upon request to assist modeling algorithm and feature cluster selection. The model score is the coefficient of determination (i.e., higher score represents better model fitting) and C) example model performance inspection for selected clusters. Each datapoint in panel C represents a single sample data.

**Table S1**. Major modules currently within *MSS* and related functions.

| Module | Function | Description |
| --- | --- | --- |
| mssmain | get_scans | Data import - HRMS data import |
|  | batch_scans | Data import - Import multiple HRMS data |
|  | noise_removal | Data reduction - HRMS data pre-processing, remove noise detections within each scan |
|  | peak_pick | Feature extraction - peak picking from imported data using defined parameters |
|  | peak_list | Feature extraction - generate a feature list based on user-defined settings |
|  | formula_prediction | Data reduction - predicting selected feature's formula with isotopic pattern scoring |
| align | mss_align | Feature alignment - align the feature lists from different samples |
|  | mss_process | Data import, feature extraction and alignment - automatic workflow from HRMS data import to MS peak list generation |
| visreader | tic_plot | Visualization - present the TIC plot for selected data |
|  | ms_spectrum/frag_plot | Visualization - present the selected spectrum indexing by numbers |
|  | ms_chromatogram | Visualization - generate a chromatogram plot for selected features |
|  | integration_plot | Visualization - generate an integrated chromatogram plot for peak area checking |
|  | iso_plot | Visualization - present the MS1 spectrum for selected features with the theoratical MS1 spectrum from input formula for comparison |
|  | overview_scatter | Visualization - provide a scatter plot of all the features within the data |
| frag | frag_massbank | Annotation - search online database for the input fragment list |
|  | frag_comp | Annotation - present the MS2 spectrum for selected precursors with optional online database search and comparison |
| dm | data_prep | Data mining - data cleaning and preliminary filters for subsequent analysis |
|  | PCA_report | Data mining - perform a PCA on selected dataset |
|  | eps_assess | Data mining - assisting tool for the DBSCAN clustering analysis |
|  | ms_cluster | Data mining - unsupervised clustering on HRMS data, dimension reduction is optional |
|  | trend_calc | Data mining - calculate the trend correlation of selected features |
|  | source_label | Data mining - tracking the source from different samples |
|  | feature_model | Data mining - modeling function towards experimental backgrounds |
|  | cluster_pred | Data mining - prediction on new data based on trained models |

**Table S2**. Key parameters for peak extraction for *MSS, MSDIAL and XCMS*

| Tools  Features | MSS | XCMS | MSDIAL |
| --- | --- | --- | --- |
| Noise removal | 2000 | 2000 | 2000 |
| Signal-to-noise threshold | 10 | 10 | NA |
| m/z error tolerance | 10 ppm | 10 ppm | 0.003 Da |
| Peak area threshold | 1e5 | 1e5 | 1e5 |
| Minimum peak width | 5 scans | 10 sec | 7 scans |

**SI References**

1. Ulrich EM, Sobus JR, Grulke CM, et al (2019) EPA’s non-targeted analysis collaborative trial (ENTACT): genesis, design, and initial findings. Anal Bioanal Chem 411:853–866. https://doi.org/10.1007/s00216-018-1435-6

2. Negri LH, Vestri C (2017) lucashn/peakutils: v1.1.0

3. Baeza-Baeza JJ, Pous-Torres S, Torres-Lapasió JR, García-Álvarez-Coque MC (2010) Approaches to characterise chromatographic column performance based on global parameters accounting for peak broadening and skewness. J Chromatogr A 1217:2147–2157. https://doi.org/10.1016/j.chroma.2010.02.010

4. Melnikov AD, Tsentalovich YP, Yanshole VV (2020) Deep Learning for the Precise Peak Detection in High-Resolution LC–MS Data. Anal Chem 92:588–592. https://doi.org/10.1021/acs.analchem.9b04811

5. Peter KT, Wu C, Tian Z, Kolodziej EP (2019) Application of Nontarget High Resolution Mass Spectrometry Data to Quantitative Source Apportionment. Environ Sci Technol 53:12257–12268. https://doi.org/10.1021/acs.est.9b04481

6. Tsugawa H, Cajka T, Kind T, et al (2015) MS-DIAL: Data Independent MS/MS Deconvolution for Comprehensive Metabolome Analysis. Nat Methods 12:523–526. https://doi.org/10.1038/nmeth.3393

7. Smith CA, Want EJ, O’Maille G, et al (2006) XCMS:  Processing Mass Spectrometry Data for Metabolite Profiling Using Nonlinear Peak Alignment, Matching, and Identification. Anal Chem 78:779–787. https://doi.org/10.1021/ac051437y

8. Hu X, Zhao HN, Tian Z, et al (2022) Transformation Product Formation upon Heterogeneous Ozonation of the Tire Rubber Antioxidant 6PPD (N-(1,3-dimethylbutyl)-N′-phenyl-p-phenylenediamine). Environ Sci Technol Lett 9:413–419. https://doi.org/10.1021/acs.estlett.2c00187

9. Zhao HN, Tian Z, Kim KE, et al (2021) Biotransformation of Current-Use Progestin Dienogest and Drospirenone in Laboratory-Scale Activated Sludge Systems Forms High-Yield Products with Altered Endocrine Activity. Environ Sci Technol 55:13869–13880. https://doi.org/10.1021/acs.est.1c03805

10. Luc P ChemCalc: A Building Block for Tomorrow’s Chemical Infrastructure | Journal of Chemical Information and Modeling. https://pubs.acs.org/doi/10.1021/ci300563h. Accessed 30 Apr 2021

11. MassBank of North America. https://mona.fiehnlab.ucdavis.edu/. Accessed 29 Sep 2021
